# Effect of moderate and regular consumption of Cinco Jotas acorn‐fed 100% Iberian ham on overall cardiovascular risk: A cohort study

**DOI:** 10.1002/fsn3.869

**Published:** 2018-10-25

**Authors:** Emilio Márquez Contreras, Ignacio Vázquez‐Rico, Agueda Baldonedo‐Suárez, Sara Márquez‐Rivero, Jesús Jiménez, Francisco Machancoses, Rocío Morano‐Báez, Antonio León‐Justel

**Affiliations:** ^1^ Clinical Management Unit Molino de la Vega Huelva Spain; ^2^ Laboratory Juan Ramón Jiménez Hospital Huelva Spain; ^3^ Clinical Management Unit La Orden Huelva Spain; ^4^ Beturia Andalusian Foundation for Health Research Juan Ramón Jiménez Hospital Huelva Spain

**Keywords:** cardiovascular disease, cholesterol, ham, nutrition

## Abstract

**Objective:**

To evaluate the impact that the moderate and regular consumption of Cinco Jotas acorn‐fed 100% Iberian ham has on overall cardiovascular risk, lipid parameters, blood pressure, and weight.

**Methods:**

A longitudinal, analytical, and quasi‐experimental clinical study with repeated measures was carried out with 100 randomly selected individuals in primary care. The sample population included men and women (64%) between the ages of 25 and 55 (42.08, *SD* 9.6) who were not diagnosed with any cardiovascular illness or diabetes, were not undergoing antihypertensive treatment, nor taking lipid‐lowering drugs. There were four visits during a 2‐week washout period for the first of three phases. Phases 2 and 3 included an 8‐week habitual diet phase followed by an 8‐week intervention phase when participants consumed 40 g daily of acorn‐fed 100% Cinco Jotas Iberian ham. Measurements of cardiovascular risk factors were taken following the SCORE table. These included total cholesterol, high‐density lipoproteins cholesterol (HDL‐c), low‐density lipoproteins cholesterol (LDL‐c), triglycerides (TG), and weight.

**Results:**

The average vascular risk (SCORE) was 0.20 (*SD* 0.49) before the consumption phase and 0.18 (*SD* 0.48) at the end of the study (*p* > 0.05). An increase in HDL‐c of 5 mg/dl was observed while there was a decrease in LDL‐c and TG of 10 mg/dl (*p* < 0.05). There were no differences in total cholesterol levels, blood pressure, or weight; nor were differences observed in average consumption of calories, protein, lipids, carbohydrates, or alcohol (*p* > 0.05).

**Conclusions:**

The daily consumption of 40 g of Cinco Jotas acorn‐fed 100% Iberian ham does not increase the risk of cardiovascular disease and has a favorable impact on lipid levels without affecting blood pressure or weight.

## 1 INTRODUCTION

Scientific evidence has proven the beneficial effect that some foods have on cardiovascular diseases (CVD), some forms of cancer, and other degenerative diseases (Deleuze Isasi, 2006), but it must also confirm that these foods do not contribute to the appearance of cardiovascular risk factors like increased cholesterol total (TC), high‐density lipoproteins cholesterol (HDL‐c), low‐density lipoproteins cholesterol (LDL‐c), triglycerides (TG), and blood pressure.

Cinco Jotas acorn‐fed 100% Iberian ham, produced principally in southwest Spain and considered a red meat, is a popular part of Spanish cuisine. This ham, made from the hind quarters of Iberian pigs nourished primarily on grass and acorns, is cured, seasoned with salt, and exposed to a range of favorable environmental conditions and habitats (Deleuze Isasi, 2006; Jiménez‐Colmenero, Ventanas, & Toldrá, 2010). The Cinco Jotas curing cellars have the right conditions to ensure proper maturation of the hams by naturally maintaining just the right temperature and humidity levels, allowing the performance of proteases found naturally in meat. This process instils in the ham certain nutritional characteristics such as high‐biological‐value proteins and elevated levels of iron, zinc, and B vitamins. Importantly, its 5% fat content is rich in unsaturated fatty acids, of which between 67% and 68% (Ortega, López‐ Sobaler, Requejo, & Andrés, 2003) is composed of oleic acid. The ham is also rich in polyphenols and amino acids which are released by the proteolysis that occurs during the curing process (De la Hoz, Lopez, Hierro, Cambero, & Ordonez, 1996).

Several studies have confirmed the cardiovascular health benefits of the Mediterranean diet and, specifically, the consumption of olive oil and other monounsaturated fats (Deleuze Isasi, 2006) associated with a lower incidence of CVD, cancer, high blood pressure, obesity, and osteoporosis (Jiménez‐Colmenero et al., 2010). Similarly, different components of this diet have been associated with providing varying degrees of protection against these same diseases (Martínez‐González & Sanchez‐Villegas, 2004; Ruiz‐Canela López et al., 2009).

The results of these studies are inconclusive, highlighting the protective health benefits of foods such as olive oil, vegetables, fruits, and nuts (Bes‐Rastrollo et al., 2007; Martínez‐González et al., 2008), while others associate the consumption of red meats with obesity, increased blood pressure, or CVD (Bes‐rastrollo et al., 2006; Dominguez et al., 2018; Kontogianni, Panagiotakos, Pitsavos, Chrysohoou, & Stefanadis, 2008; Ruiz‐Canela López et al., 2009; Wang, Manson, Buring, & Sesso, 2008; Xu, Yin, & Tong, 2007). In the case of the latter, occasional consumption is recommended due to the high content of saturated fatty acids, although it must be considered that different red meats have varying nutritional compositions and effects on one's health (Ruiz‐Canela López et al., 2009).

As a result, the moderate and regular consumption of Cinco Jotas acorn‐fed 100% Iberian ham can be considered to have beneficial effects on cardiovascular health. Several studies have addressed this line of research. While it is true that these studies are not conclusive due to population biases and small sample populations, atherothrombotic profile improvements were observed (Maciá Botejara et al., 2005), as were improvements to lipid profile (Martín, Escudero‐Gilete, Romero, & Vicario, 2009; Mayoral et al., 2003; Rebollo et al., 1998). Nondetrimental effects on CVD, blood pressure, and weight gain were also observed (Ruiz‐Canela López et al., 2009).

The objectives of this study were primarily to evaluate the impact that the moderate and regular consumption Cinco Jotas acorn‐fed 100% Iberian ham has on overall cardiovascular health and, secondarily, on lipid parameters (TC, TG, HDL‐c, and LDL‐c), blood pressure, weight, and waist circumference (WC).

## 2 MATERIAL AND METHODS

This clinical study was designed to be longitudinal, analytical, and quasi‐experimental with pre‐ and postrepeated measures. With a level of significance of 5%, assuming that the proportion in the reference group is 80.00%, and the proportion in the experimental group is 90.00%, and that the superiority limit is 2.00%, we needed to include 97 subjects in the study. Estimating a 9% dropout rate, a recruit total of 106 participants was deemed appropriate. The software Ene 3.0 was used to estimate the size of the sample population. The participants were randomly selected from patients who had visited the Health Centre during the last three weeks of June 2016 using numbers randomly generated from Microsoft Excel. Six subjects were selected from the daily roster of patients for each doctor. If they met the criteria for inclusion, they were invited to participate in the study. Approximately 60 subjects were recruited each week.

The inclusion criteria were as follows: (a) individuals of both sexes between the ages of 25 and 55 years old; (b) absence of any documented CVD (including stroke, transient ischemic attack, acute myocardial infarction, acute coronary syndromes, stable ischemic cardiopathy, or peripheral arterial disease); (c) individuals who have provided written informed consent; (d) individuals whose caloric intake was between 80 and 105% of the daily amount recommended for their profile and met the nutritional objectives for the general adult population regarding macronutrients. The criteria for exclusion were as follows: (a) pregnant or breastfeeding women; (b) individuals whose pathological situation could interfere with the development of the study(e.g., acute stroke, acute myocardial infarction or cancer); (c) individuals who were already participating in any other study; (d) individuals undergoing pharmacological treatment for high cholesterol or high blood pressure; and (e) diabetics.

Subjects could be withdrawn from the study if they developed any illness that would complicate their participation in the study, or if the subject decided not to continue with the study or the prescribed visits.

The study took place between June and November 2016 with each subject monitored for 18 weeks. Nine primary care investigators (five doctors and four nurses) and two laboratory technicians participated in this study.

The study was composed of three phases:

1. Washout phase: A 2‐week period during which subjects were to continue their usual dietary habits, taking care not to change their eating behaviors.
2. Habitual diet phase: An 8‐week period during which subjects were to continue their usual dietary habits. This phase was controlled by a diet survey.
3. Intervention phase: The individuals received 40‐g portions of vacuum‐packed Cinco Jotas acorn‐fed 100% Iberian ham to eat daily at breakfast for 8 weeks. The check‐in visits were attended by a nurse in the health center who was experienced with the food survey and in the extraction, preparation, and distribution of samples. The subjects were advised to continue their usual diet throughout the study. Compliance with the daily intake of the product was assessed by counting empty packages, with a 90% compliance considered to be compliant. The product provided contained Cinco Jotas acorn‐fed 100% Iberian ham, sea salt, antioxidant E‐301 (sodium ascorbate), acidity regulator E‐331(trisodium citrate), and preservative E‐252 (potassium nitrate).E‐301, E‐331, and E‐252 have a technical function during the initial phase of production but by the end of the process they deteriorate and are no longer present in the final product. The quantities added are negligible, such minute quantities that they do not affect the final amount of ions in the end product.

The results of a nutritional study based on 54 samples are shown in Table 1.

**Table 1:** Nutritional profile of Cinco Jotas acorn‐fed 100% Iberian ham, calculated from a sample of 54 packs of ham used during the study

Fat of which (g/100 g)	26.34 (2.83)	Riboflavin (mg/kg)	6.41 (0.44)
Saturated fatty acid (g/100 g)	33.51 (1.04)	Nicotinamide (mg/kg)	36.42 (3.46)
Polyunsaturates fatty acid (g/100 g)	7.98 (0.24)	Pantothenic acid (mg/kg)	18.25 (3)
Monounsaturates fatty acid (g/100 g)	58.46 (1.71)	Pyridoxine (mg/kg)	3.46 (0.47)
Oleic acid (g/100 g) of total fat	54.26 (0.58)	Biotin (μg/100 g)	0 (0)
Moisture (g/100 g)	31.54 (2.74)	Folic acid (μg/100 g)	0 (0)
Carbohydrates (g/100 g)	0.52 (0.21)	Cyanocobalamin (μg/100 g)	0 (0)
Protein (g/100 g)	35.79 (2.14)	Vitamin E (μg/100 g)	2.86 (0.67)
Ash (g/100 g)	5.81 (0.33)	Calcium (mg/kg)	54.84 (5.83)
Sodium (g/100 g)	1.70 (0.12)	Zinc (mg/kg)	44.02 (3.41)
Salt (g/100 g)	4.24 (0.31)	Magnesium (mg/kg)	319.58 (17.46)
Iron (mg/kg)	27.64 (2.48)	Potassium (mg/100 g)	5852.75 (1922.45)
Calories (energy value) (kcal/100 g)	382.30 (23.11)	Phosphorus (mg/kg)	2588 (281.60)
Thiamine (mg/kg)	3.46 (0.57)	Selenium (mg/kg)	0.10 (0)

*Note*

Data were expressed as an average and standard deviation (*SD*).

The 40‐g portions of ham were sent by courier weekly from the slicing and packing room of the producer in Jabugo to the health center, where they were collected by the subjects. The portions did not require refrigeration because they were delivered within 5 days of consumption. The cured ham was provided sliced, in 40‐g portions, in thermoforming vacuum packaging, without identifying the brand on the packaging.

There were four visits, the initial visit and three follow‐up visits at 2, 10, and 18 weeks. During the initial screening visit, criteria were reviewed to confirm subject eligibility, participants were fully briefed, both orally and in print form, and they signed informed consent forms. Each subject provided their clinical history and the SCORE table was used to evaluate levels of cardiovascular risk. Weight, height, WC, and blood pressure were measure at each visit. Blood pressure was taken using an Omron M6 blood pressure monitor. The recommendations of the American Heart Association were followed; thus, the anthropometric measurements (weight and height) were taken with participants barefoot and wearing light clothes, using a stadiometer and a scale with a precision of 100 g, and the WC was recorded using a calibrated measuring tape.

The Spanish Food Composition Database (http://www.bedca.net/bdpub) was used to determine the caloric and nutrient content of the food. To determine if the patient had followed a stable daily diet, the nutritional survey of the Community of Madrid (ENUCAM) was administered both at the beginning and at the end of the study. This survey consists of two subsurveys; the first is a 24‐hour dietary recall survey administered by a nurse. It records the times and content of meals or snacks ingested during the previous day (food and beverages), including quantities, ingredients, and preparation methods. The second part was a self‐administered survey about the participant's own consumption of food during the previous week. The recommendations and clinical procedures used by our reference laboratory at the Juan Ramón Jiménez Hospital in Huelva were used for the measurement of biochemical parameters.

The main variable assessed was cardiovascular risk, for which the SCORE table was used to combine factors such as age, gender, smoker/nonsmoker (33% of the sample were smokers), systolic blood pressure (SBP), and TC level. The secondary variables were TC level, HDLc, LDL‐c, TG, glycemie, uric acid (UA), C‐reactive protein (CRP), weight, and WC. The diet, caloric and nutritional intakes of each individual and physical activity were calculated using the ENUCAM surveys. The detection of biochemical markers was carried out in the Juan Ramón Jiménez Hospital laboratory in Huelva, and the preparation of the Cinco Jotas acorn‐fed 100% Iberian ham sample in the Cinco Jotas laboratory of Jabugo in the Huelva province of Spain.

A univariate analysis of both socio‐demographic and clinical variables was first performed to describe the variables in each participant sample. Subsequently, a bivariate analysis was performed to measure the difference (if any) between the values in the pre‐ and postanalysis groups. The main variable was evaluated by calculating the mean decreases in each phase and comparing the mean decreases of the SCORE measures. Both the repeated‐measures ANOVA test and the Wilcoxon signed‐rank test were used, including their respective effect size estimates: partial eta‐squared (η^2^) for the ANOVA and Cohen's *d* for the Wilcoxon signed‐rank test. In all cases, a statistical significance of 5% was required (*p* < 0.05), thus determining the confidence intervals. The statistical analyses were carried out by the Methodology Department of the Beturia Foundation for Health Research using the statistical program SPSS version 21.0.

## 3 RESULTS

A total of 148 subjects were evaluated, of which 105 were recruited. Forty‐three individuals were excluded from the study; 22 were over the age of 55 (17 women and five men), one was pregnant, three women and two men presented multiple pathologies, three women declined to participate, and 12 presented with high blood pressure, diabetes, or dyslipidemia under pharmacological treatment (seven women and five men).

Of the 105 subjects eventually selected, 100 completed the study: one subject moved away from the area, two did not want to repeat the periodic visits to the center for analysis, and two cited family problems that prevented them from continuing. The final sample was composed of 64 women (64%) and 36 men (36%) with a global average age of 42.08 (*SD* 9.6) years.

The compliance with the daily intake of ham was 92%. The remaining 8% reported that although they had discarded the packaging, they had in fact eaten the daily portion. The average cardiovascular risk was calculated as 0.20 (*SD*: 0.49) before the intervention phase and 0.18 (*SD*: 0.48) at the end of the study, with no significant differences observed (*p* > 0.05, *d*_C_ = 0.04, *r*_Yλ_ = 0.02).

Table 2 (Supporting Information) shows the exploratory findings and the analytical results, which follow a normal distribution according to the Kolmogorov–Smirnov test, so an ANOVA test of repeated measures was performed. Significant differences were observed in the means, with an increase in HDL‐c and a decrease in LDL‐c, TG, and UA.

**Table 2:** Biochemical parameters of the samples, systolic and diastolic blood pressure, weight, and waist circumference

	Visit 1	Visit 2	Visit 3	*F*	*p*	$\eta_{p}^{2}$	1–β
TC (mg/dl)	184.8 (3.3)	189.2 (3.3)	186.3 (3.2)	2.634	0.077	0.054	0.512
HDL‐c (mg/dl)	57.8 (1.4)	58.6 (1.4)	64.1 (1.6)	29.111	0.000	0.385	1.00
LDL‐c (mg/dl)	93.8 (4.5)	98.8 (4.7)	88.4 (4.6)	5.893	0.004	0.114	0.866
TG (mg/dl)	92.6 (4.5)	97.7 (4.6)	87.3 (4.5)	4.729	0.011	0.092	0.778
G (mg/dl)	84.4 (8.7)	84.6 (9.8)	84.4 (9.2)	0.070	0.933	0.001	0.060
CR (mg/dl)	0.75 (0.16)	0.76 (1.5)	0.76 (0.16)	0.041	0.960	0.001	0.056
UA (mg/dl)	4.3 (1)	4.5 (1.1)	4.3 (1)	4.501	0.014	0.098	0.755
CRP (mg/dl)	2.8 (5.3)	2.6 (3.7)	2.3 (2.6)	0.484	0.618	0.011	0.127
SBP (mmHg)	122.3 (1.3)	121.4 (1.4)	122.3 (1.4)	1.013	0.367	0.020	0.222
DBP (mmHg)	74.5 (0.7)	74.3 (0.8)	74.5 (0.7)	0.119	0.888	0.002	0.068
Weight (kg)	71.3 (15.8)	72.5 (15.5)	72.3 (15.5)	1.894	0.156	0.037	0.385
WC (cm)	85.7 (12.7)	85.5 (12.3)	85.1 (12.1)	3.289	0.051	0.063	0.612

*Notes*

TC: Total cholesterol; HDL‐c: high‐density lipoprotein; LDL‐c: Low‐density lipoproteins cholesterol; TG: triglyceride level; G: glycemie; CR: creatinine; UA: uric acid; CRP: C‐reactive protein; SBP: systolic blood pressure; DBP: diastolic blood pressure; WC: waist circumference.

Data were expressed as an average and standard deviation (*SD*).

Table 3 shows the average daily consumption of kilocalories, macronutrients, and alcohol according to the 24‐hr recall and self‐administered surveys. As these variables did not follow a normal distribution according to the Kolmogorov–Smirnov test, we applied the Wilcoxon signed‐rank test and calculated the Cohen's *d* to measure effect size.

**Table 3:** Average consumption of energy, macronutrients, and alcohol

	24‐hr recall survey					Self‐administered survey				
*N* = 100	Initial	Final	*p*	*d*	*r*_Yλ_	Initial	Final	p	d	r_Yλ_
Energy (Kcal) Calories	2258.4 (496.1)	2282.8 (461.5)	0.420	−0.05	−0.02	2262.4 (498.1)	2272.8 (455.8)	0.514	−0.02	−0.01
Proteins (g)	98.7 (31.4)	99.8 (32.9)	0.664	0.03	0.02	95.7 (32.1)	97.8 (32.8)	0.363	−0.06	−0.03
Lipids (g)	94.4 (31.1)	93.0 (28.3)	0.640	0.04	0.02	94.5 (31.0)	93.0 (28.2)	0.563	0.05	0.02
CHO (g)	233.0 (89.8)	233.4 (76.9)	0.530	−0.00	−0.00	233.0 (89.8)	236.3 (80.0)	0.730	−0.04	−0.02
Alcohol (g)	3.0 (5.5)	2.9 (5.9)	0.737	0.02	0.01	3.2 (5.8)	2.5 (5.1)	0.096	0.13	0.06

*Note*

Data were expressed as an average and standard deviation (*SD*). CHO: Carbohydrates.

No significant differences were observed during active intervention, between the values obtained in the physical activity survey in the type of work developed, light, active, or very active. Seventy‐four percent and 77% report performing some type of physical activity, without significant differences. Highlights as 23% and 22% perform physical activity daily, and as 26 and 23% perform physical activity less than once a month. No significant differences were observed between the performance of another physical or sports activity during active intervention.

## 4 DISCUSSION

Based on the results obtained, it can be concluded that Cinco Jotas acorn‐fed 100% Iberian ham can be consumed daily without any risk to cardiovascular health since it did not increase the risk in individuals consuming a daily portion of 40 g, a portion that can be considered regular and acceptable. No significant changes were observed in the creatinine (CR), blood pressure, TC levels, glycemie, CRP, weight, and WC; values that did not change throughout the study.

These results coincide with those obtained by Maciá Botejara et al. (2005), who observed an improvement in the atherothrombotic profile, and those of Mayoral et al. (2003), who observed a drop in blood pressure, increase in antioxidants, and decrease in lipid peroxidation after a period of 6 weeks consuming cured ham made from acorn‐fed 100% Iberian pigs.

Significant differences have been observed in the composition of some fatty acids in pigs due to differences in the feeding plan (Bes‐Rastrollo et al., 2006; Bes‐Rastrollo et al., 2007; De la Hoz et al., 1996; Dominguez et al., 2018; Estruch, Ros, Salas‐Salvad, Covas, & Corella, 2013; Kontogianni et al., 2008; Maciá Botejara et al., 2005; Martínez‐González & Sanchez‐Villegas, 2004; Martínez‐González et al., 2008; Ruiz‐Canela López et al., 2009; Wang et al., 2008; Xu et al., 2007), with favorable outcomes for animals who graze free range during the montanera season, as is the case with Cinco Jotas acorn‐fed 100% Iberian ham. Animals were fed diets of grass and acorns (montanera), acorns and cereals (recebo), or cereals (cebo). The total lipid fraction from intramuscular fat resulted in significant differences (*p* ≤ 0.05) between all three batches in the composition of C18:1 and C 18:2, and between montanera and the other two batches in the fatty acids C14:0 and C16:0. For intermuscular fat, significant differences (*p* ≤ 0.05) were observed between all three batches for the fatty acids C14:0, C16:0, C18:1, C18:2, and C18:3). Although Cinco Jotas acorn‐fed 100% Iberian ham can be considered a red meat, our results indicate that its habitual consumption cannot be associated with obesity, nor with the increase in blood pressure or CVD, as happens with the habitual consumption of other red meats (Bes‐rastrollo et al., 2006; Kontogianni et al., 2008; Ruiz‐Canela López et al., 2009; Wang et al., 2008; Xu et al., 2007). This may be due to the nutritional characteristics linked to the curing process (De la Hoz et al., 1996; Ortega et al., 2003), as previously indicated. On the contrary, the study results show that consuming Cinco Jotas acorn‐fed 100% Iberian ham as part of a Mediterranean diet can be considered to be heart healthy, producing an increase in HDL‐c and a decrease in LDL‐c, TG, and UA. These results agree with those of Martín, Escudero‐Gilete, Romero, and Vicario (Martín et al., 2009). In fact, as already mentioned, several studies point out the beneficial effects of the Mediterranean diet and the consumption of monounsaturated fats (Martínez‐González & Sanchez‐Villegas, 2004; Ruiz‐Canela López et al., 2009). The Cinco Jotas ham used in the study contains a high concentration of monounsaturated fatty acids as well as other foods included in the Mediterranean diet.

Saban‐Ruiz et al. (2017) conducted a study evaluating the arterial endothelium in 50 healthy subjects between 25 and 55 years of age, who consumed 50 g of ham daily for 6 weeks. In this study, they compared the effects of the consumption of Serrano ham and acorn‐fed Iberian ham, observing that the regular consumption of both was associated with cardiovascular benefits. Although both types of ham produced an improvement in endothelial function, this improvement was superior in the case of the acorn‐fed Iberian ham. This could be explained by its higher content of polyphenols, potent antioxidants, and anti‐inflammatory effects at the vascular level. Additionally, neither of them produced weight gain, nor did they modify the lipid profile. Moderate decreases in blood pressure were also observed. These results support those found in this current study.

Multiple studies have identified natural peptides with antihypertensive effects generated during the curing process of Spanish dry‐cured ham (Escudero, Aristoy, Nishimura, Arihara, & Toldrá, 2012; Escudero et al., 2013; Martínez‐Sánchez et al., 2017; Mora, Escudero, Arihara, & Toldrá, 2015). These peptides are powerful antioxidants and inhibitors of the angiotensin converting enzyme (ACE), useful in counteracting the hypertensive effect of the high salt content (Ruiz‐Canela López et al., 2009).

Beyond phenolic compounds, other phenomena could help maintain blood pressure, such as the high content of oleic acid and proteins or the increased bioavailability of nitric oxide for endothelial improvement and its vasodilator effect, together with the decrease in the vasoconstriction factors of the vascular bed (Martínez‐Sánchez et al., 2017).

Several limitations of the present study should be considered. The sample consisted of young and healthy subjects, without including risk or clinical groups. The sample obtained from 100 individuals has been sufficient to achieve the objectives of the study. It has been obtained randomly among patients under 55 years of age who attend a health center, present no cardiovascular risk factors, and complied with the prescribed intake to a high degree. Therefore, the results can be applicable to a similar population. A future study could involve a comparison with these alternative groups, in order to analyze the effect of Cinco Jotas acorn‐fed 100% Iberian ham consumption on an at‐risk or sick population. The use of surveys to track food consumption and physical exercise, although validated by other studies, may also be subject to social bias, since the data obtained are based on the recall or journal‐keeping habits of the subjects themselves. This stumbling block is difficult to overcome, and although a study could be done under controlled feeding conditions, it would then lose ecological validity.

In conclusion, consuming 40 g of Cinco Jotas acorn‐fed 100% Iberian ham daily is recommended for the general population. It does not increase cardiovascular risk, has favorable effects on different lipid parameters, and does not affect blood pressure or weight. It could be recommended for dyslipidemic patients with low HDL, where effective therapeutic treatments are scarce. However, more evidence is necessary.

## AUTHORSHIP

All authors should have made substantial contributions to all of the following: Dr. Márquez Contreras E; Dr. Vázquez‐Rico, I; Dr. Baldonedo‐Suárez, A; Dr. Márquez‐Rivero, S; Dr. León‐Justel, A; Mr.Machancoses, F; Mr Morano‐Báez, R.: involved in the conception and design of the study. Dr. Márquez Contreras E; Dr. Vázquez‐Rico, I; Dr. Baldonedo‐Suárez, A; Dr. Márquez‐Rivero, S Mr. Jiménez, J.: acquired of data. Dr. Márquez Contreras E; Dr. Vázquez‐Rico, I; Dr. Baldonedo‐Suárez, A; Dr. Márquez‐Rivero, S; Dr. León‐Justel, A; Mr.Machancoses, F; Mr Morano‐Báez, R.: analyzed and interpreted the data. Dr. Márquez Contreras E; Dr. Vázquez‐Rico, I; Dr. Baldonedo‐Suárez, A; Dr. Márquez‐Rivero, S; Dr. León‐Justel, A.: involved in drafting the article or revising it critically for important intellectual content. All authors: involved in final approval of the version to be submitted.

## CONFLICT OF INTEREST

There is no conflict of interest. The authors declare that they have no conflict of interests in the research. The study was sponsored through a research grant from Osborne Wineries that financed the samples of Iberian ham (Osborne Wineries had no role in the design, analysis, or writing of this article). The lead author affirms that this manuscript is an honest, accurate, and transparent account of the study being reported. The reporting of this work is compliant with STROBE guidelines. The lead author affirms that no important aspects of the study have been omitted and that any discrepancies from the study as planned.

## ETHICAL STATEMENT

This study was performed in accordance with the ethical principles laid down in the Declaration of Helsinki for human subjects. This study was evaluated, reviewed, and approved by the Ethical Committee of Juan Ramón Jiménez Hospital of Huelva (Spain). Written informed consent was obtained from all study participants.
